# Shear Stress Promotes Metastasis of Triple-negative Breast Cancer Cells Through Calcium Channel-ROS-FOS Axis

**DOI:** 10.7150/ijbs.127645

**Published:** 2026-04-08

**Authors:** Huaxing Xiong, Muya Zhou, Kathy Qian Luo

**Affiliations:** 1 Department of Biomedical Sciences, Faculty of Health Sciences, University of Macau, Taipa, Macao SAR 999078, China.; 2 Ministry of Education Frontiers Science Center for Precision Oncology, University of Macau, Taipa, Macao SAR 999078, China.

**Keywords:** shear stress, reactive oxygen species, TNBC metastasis, calcium channel, FOS

## Abstract

During metastasis, circulating tumor cells (CTCs) are subjected to fluidic shear stress (SS), which eliminates many of them but paradoxically enhances malignancy and metastatic success. Meanwhile, given the critical roles of reactive oxygen species (ROS) in stress response and cancer, we engineer a circulation-mimicking microfluidic system which generates pulsatile SS to investigate the interplay among SS, ROS and metastasis. A 3-hour SS treatment rapidly elevates ROS levels, boosting metastatic abilities in triple-negative breast cancer (TNBC) cells *in vitro* and* in vivo*. RNA-sequencing and subsequent investigation identify activator protein-1 (AP-1) transcription factor members FOS, ATF3 and FOSB, which undergo dramatic ROS-dependent increase and nuclear localization upon SS stimulation. All three genes exhibit metastasis-promoting potential, while FOS displays the strongest ability to trigger distant lung metastasis in an orthotopic tumor model and correlates with worse clinical outcomes. Mechanistically, calcium channel acts as the mechano-sensor to initiate the SS-ROS cascade, with calcium channel blockers Mibefradil and Nifedipine effectively weakening SS-ROS-induced invasiveness. Following ROS elevation, the downstream activation of p38-ELK1-cFOS and JNK-cJUN pathways subsequently increase the expression of malignancy-related genes. This metastasis-promoting SS-calcium channel-ROS-FOS axis provides new insights for combating metastatic progression in breast cancer.

## Introduction

Triple negative breast cancer (TNBC) is the most malignant breast cancer sub-type, with a higher rate of distant metastasis and worse clinical outcomes 1,2. Metastasis has long been considered as the most common cause of cancer-related deaths, but it is actually a multi-step process that includes detachment from the primary tumor, intravasation and circulation in the blood vessels, extravasation and colonization at secondary sites 3,4. As an essential but challenging step of metastasis, circulation eliminates many of the cancer cells through fluid shear stress (SS) and immuno-surveillance, and only those cells with strong malignancy successfully disseminate to distant organs 5,6. Such notion that malignant circulating tumor cells (CTCs) are the origin of metastasis highlights the importance of understanding how cancer cells become invasive under the circulatory environment.

Being a mechanical force generated from blood flow, SS has been shown to induce apoptosis of CTCs via apoptotic agent-death receptor pathway or by damaging mitochondria, thus acting as a barrier for preventing metastasis 7,8. However, emerging evidence has been indicating its metastasis-promoting potential where, at the same time, cancer cells display molecular changes and acquire malignant phenotypes in response. For example, in prostate cancer, Piezo1 sensed SS and facilitated metastasis via downstream Src signaling 9; desmosomal proteins dsmocolin-2 and plakophilin-1 were elevated in SS-resistant breast and lung cancer cell sub-populations, allowing them to form clusters, survive SS and further metastasize 10, while SS-induced cleavage of protease-activated receptor 2 (PAR2, a G-protein coupled receptor (GPCR)) by mesotrypsin initiated downstream pathway and promoted metastasis of lung cancer cells 11. The distinct performance and outcomes of cancer cells under SS call for investigations to unravel the underlying mechanisms.

To study the interactions between SS and cells, researchers have developed several experimental models. For instance, a syringe pump-based model could generate high levels of SS up to 6,400 dynes/cm^2^ on cancer cells 12, while a Darcy flow apparatus generated low levels of SS ranging from 0.1 to 0.6 dynes/cm^2^ on collagen-embedded glioma cells 13, and a shear flow system allowed scientists to assess the effects of SS on endothelial cell monolayer 14. Previous work by our colleagues has engineered a peristaltic pump-based microfluidic system to study the impact of SS on cancer cells* in vitro* 15. The peristaltic pump generated pulsatile shear flow to mimic the vascular SS, and the soft-materialled silicone tubing provided an environment similar to the circulation system in human body. By applying this system, our colleagues showed that 6-h arterial SS treatment significantly elevated reactive oxygen species (ROS) in a metastatic TNBC cell line MDA-MB-231, and following-up research demonstrated ROS-dependent increase of migration and adhesion abilities via phosphorylation of extracellular signal-regulated kinases (ERK) and focal adhesion kinase (FAK) 15-17. Indeed, existing knowledge indicates the essential roles of ROS in cancer progression, where they act as important signaling molecules for massive cancer-related networks 18, thereby highlighting a critical aspect that explores the interplay between SS, ROS and diseases. Moreover, mechano-transduction allows cells to convert mechanical force into signaling molecules and have been well studied in vascular endothelial cells, where mechano-sensors plays critical roles under SS stimulation 19,20. In the context of circulating cancer cells, though some mechano-sensors have been identified (for instance, the aforementioned Piezo1 and PAR2), to date the mechanisms of mechano-transduction are still partially understood. In particular, the SS-ROS-metastasis cascade in TNBC cells remains to be investigated.

Therefore, with the aim of elucidating how TNBC cells sense SS, produce ROS and alter their molecular signatures in response to acquire malignant phenotypes, we here apply our engineered microfluidic circulatory system to explore the earlier events in TNBC cells in response to SS stimulation, with particular focus on mechano-sensing and the downstream signaling pathway after ROS elevation. Our findings will detail the mechanisms and help fill in the blanks of our previous SS-ROS-metastasis axis in TNBC cells, thereby providing foundation for developing metastasis-targeting therapies.

## Materials and Methods

### Cell lines and cell culture

Human TNBC cell line MDA-MB-231 (Research Resource Identifier (RRID): CVCL_0062, Cellosaurus) was obtained from the American Type Culture Collection (ATCC) and cultured using Dulbecco's Modified Eagle Medium (DMEM; #12100046, Thermo Fisher Scientific, USA). The human TNBC cell line BT549 (RRID: CVCL_1092, Cellosaurus) was given by Prof. Lijun Di at the Faculty of Health Sciences at the University of Macau, Macau, China and was cultured with Roswell Park Memorial Institute (RPMI) 1640 culture medium (#31800022, Thermo Fisher Scientific, USA). Both cell lines were supported with 10% Fetal Bovine Serum (FBS; #A52567-01, Gibco, USA) and 1% penicillin-streptomycin (PS; #15140122, Thermo Fisher Scientific, USA) in the culture media.

### Reagents

Antioxidants propyl gallate (PG; #48710, Sigma-Aldrich, Germany), N-acetyl-L-cysteine (NAC; #A9165, Sigma-Aldrich, Germany); H_2_O_2_ (#31642, Sigma-Aldrich, Germany); Mibefradil dihydrochloride (#HY-15553A, MCE); diphenylene iodonium chloride (DPI) (#D2926, Sigma-Aldrich, Germany); Rhapontigenin (#HY-N2229, MCE); Rhosin hydrochloride (#HY-12646, MCE); p38 inhibitor SB202190; JNK inhibitor SP600125; Nifedipine (HY-B0284, MCE) were purchased from relevant distributors.

### Microfluidic circulatory system

The microfluidic circulatory system was developed previously 15. Briefly, this system was based on a peristaltic pump (Ismatec, Germany) which generated pulsatile SS, with a cotton-filtered reservoir to minimize air-borne contaminations and culture media evaporation, and a connecting tube (#MFLX95714-18, Ismatec, Germany) and a 1.5-meter-long silicone tubing (radius 250 μm; #06411-59, Cole Parmer, Illinois). The intensity of SS was calculated using the Poiseuille's equation: τ* =* 4*Qη/πR^3^*, where τ was the SS level (dyne/cm^2^), *Q* was the flow rate (cm^3^/s), *η* was the dynamic viscosity of the culture medium (0.012 dynes/cm^2^),* π* was the mathematical constant of the ratio of a circle's circumference to its diameter, and *R* was the radius of the silicone tubing (250 μm). SS at 15 dynes/cm^2^ represented the average arterial SS in human 12 and was used in this study.

To prepare the microfluidic system, the system was sequentially: a) sterilized with 70% ethanol; b) washed with Milli-Q water for 3 times; and c) coated with 1% Pluronic F-127 (#P2443, Sigma-Aldrich, Germany) to minimize adhering of cells to the tubing. Cancer cells were trypsinized from culture, re-suspended at 20 × 10^4^ cells/mL in suitable culture medium, and 1 mL cell suspension was injected to the system via the reservoir and circulated under certain conditions. Afterwards, cells were collected for further experiments.

### ROS and Ca^2+^ detection

After SS treatment or trypsinization from culture, cells were collected, washed with phenol-red-free DMEM (#21063, Gibco), and stained with 5 μM CM-H_2_-DCFDA (#C6827, Thermo Fisher Scientific, USA) (for detecting ROS), or 2 μM X-Rhod-1 AM (#X14210, Thermo Fisher Scientific, USA) (for detecting Ca^2+^) for 15 min at 37 ℃. Cells were then washed with phenol red-free DMEM, re-suspended and imaged using fluorescence microscope (Carl Zeiss, Axio Observer Z1, Germany). Cellular ROS and Ca^2+^ levels were calculated as the average fluorescent intensity per cell using OpenCV and ImageJ.

### Transwell migration and invasion assays

After being collected from certain conditions, 1 × 10^4^ MDA-MB-231 cells or 5,000 BT549 cells in 100 μL serum-free culture medium were seeded into the Transwell inserts (#3422, Corning, USA), meanwhile 600 μL medium supported with 10% FBS was added to the lower chamber, followed by an 18-h incubation at 37 ℃. For invasion assay, the Transwell inserts were pre-coated with 100 μL Matrigel (#356230, Corning, USA), which was diluted 1 in 30 with serum-free culture media, for 2 h at 37 ℃ to form Matrigel layer before seeding cells. Afterwards, cells remained on the upper side of the Transwell chamber were removed by a cotton swab and the migrated or invaded cells on the bottom side were fixed with 4% paraformaldehyde (PFA; #158127, Sigma-Aldrich, Germany) for 15 min and stained with 0.5% Crystal Violet (#C6158, Sigma-Aldrich, Germany) for 15 min. The Transwell membranes were then cut from the inserts and mounted onto glass slide using DPX mountant (#06522 Sigma-Aldrich, Germany). Images were taken using bright field microscopy (M165FC, Leica, Germany) and quantified using ImageJ.

### Colony formation assay

Cells were collected from certain treatment, and 1,000 cells were seeded into each well of 6-well plates in normal culture media and allowed to grow for 7 days for MDA-MB-231 and 10 days for BT549 cells. Afterwards, the colonies were fixed with 4% PFA for 15 min and stained with 0.5% Crystal Violet for 15 min. Images were taken using bright field microscopy (MVX10, Japan) and quantified using ImageJ.

### MTT assay

Cells were collected from designed experimental conditions, and 100 μL cells were seeded into 96-well plates, followed by 4-h incubation with 10 μL MTT solution (#M2128, Sigma-Aldrich, Germany) and addition of 100 μL 10% SDS with 0.01 M HCl for overnight. The absorbance was measured at 595 nm by Plate Reader (PerkinElmer VICTORX3, USA).

### RNA-sequencing analysis

MDA-MB-231 cells were collected after trypsinization or SS treatment for 3 h and suspended in TRIzol (#15596026, Thermo Fisher Scientific, USA). RNA-sequencing was performed by Novogene (China). Analyses were conducted by Novogene and using R equipped with clusterProfiler 21, VolcaNoseR 22 and Python.

### RNA extraction and RT-qPCR

Total cellular RNA was extracted using TRIzol followed by reverse transcription using iScript complementary Deoxyribonucleic acid (cDNA) synthesis kit (#1778890, Bio-Rad, USA) and C1000 Thermal Cycler (Bio-Rad, USA). qPCR was conducted using iTaq Universal SYBR Green (#1725122, Bio-Rad, USA) with C1000 Thermal Cycler (real-time system) (Bio-Rad, USA) and relative messenger RNA (mRNA) levels were determined by comparing with glyceraldehyde-3-phosphate dehydrogenase (GAPDH). The primers used are listed in Table S1.

### Western blotting

Cells were lysed in radioimmunoprecipitation assay (RIPA) buffer with 1% protease inhibitor and phosphatase inhibitor cocktail (Sigma-Aldrich, Germany) and proteins were extracted and quantified using Bio-Rad protein assay dye (#5000006, Bio-Rad, USA). Proteins were equally loaded and separated by Sodium dodecyl-sulfate polyacrylamide gel electrophoresis (SDS-PAGE) and transferred onto nitrocellulose membrane (Bio-Rad, USA). The membrane was then blocked with 5% blotting-grade blocker (#1706404, Bio-Rad, USA) and incubated with specific primary antibody overnight at 4 ℃. Afterwards, the membrane was incubated with corresponding HRP-conjugated secondary antibodies at room temperature for 1 h. Finally, the proteins were visualized using Clarity Western ECL Substrate (#1705061, Bio-Rad, USA) and imaged with ChemiDoc Touch Imaging System (Bio-Rad, USA). Data was analyzed using Image Lab Software (Version 3.0 Beta 3, Bio-Rad, USA). The antibodies used are listed in Table S2.

### Small interfering RNA (siRNA)/short hairpin RNA (shRNA)-mediated gene knockdown and gene overexpression

The si/shRNA sequences were designed according to their hydrogen bond index 23 to achieve better knockdown efficiencies. All siRNAs were purchased from General Biol (China), all shRNAs and overexpression vectors were purchased from VectorBuilder Company (China). The sequences are listed in Table S3 to S5.

### Immunofluorescence

Sterilized glass slides were coated with poly-D-lysine hydrobromide (Sigma-Aldrich) for cells to adhere. Cells were collected and seeded onto the coated coverslips and allowed to attach for 30 min at 37 ℃, sequentially followed by 15-min fixation with 4% PFA, 15-min permeabilization with 0.2% Triton X-100 (Sigma-Aldrich), and 1-h blocking with 3% bovine serum albumin (BSA) (BioFroxx, Germany). Afterwards, the cells were incubated with primary antibodies at 4 ℃ overnight and secondary antibodies for 1 h at room temperature. The nuclei were stained with Hoechst 33342 (Thermo Fisher Scientific). Images were captured using confocal microscope (Confocal LSM710, Carl Zeiss) and analyzed with ImageJ and Python.

### Lung colony formation assay and orthotopic mammary fat pad xenografts

All experiments using animal models were approved by the University of Macau Animal Ethics Committee (approved protocol IDs: UMARE-025-2017 and UMARE-026-2017). For lung colony formation assay, 30 × 10^4^ cancer cells in 100 μL 1× potassium buffered saline (PBS) were injected through the tail vein of female non-obese diabetic/severe combined immunodeficiency (NOD/SCID) mice (6 to 8 weeks' old). Mice were sacrificed at 7- or 28-days post injection, and lungs were dissected and imaged with Olympus fluorescence microscope (MVX10, Japan). In orthotopic mammary fat pad xenograft model, 200 × 10^4^ cancer cells were injected into each side of the fourth pair of mammary fat pads, and tumor volumes and body weights were monitored weekly. Mice were sacrificed 42 days post injection, and tissues (tumors, iliac lymph nodes, lungs) were imaged using Olympus fluorescence microscope (MVX10, Japan).

### Kaplan-Meier survival analysis

The Kaplan-Meier survival analysis of overall survival (OS) and post-progression survival (PPS) of breast cancer patients were performed using KM-Plotter 24. Parameters were set as: split patients by upper tertile; ER- (IHC/array), PR- (IHC), HER2- (array) (OS); Grade I, II or III (PPS).

### Immunohistochemistry (IHC)

Clinical TNBC samples were purchased from Superchip ltd. (China). The samples were deparaffinized and rehydrated using Leica ST5020-CV5030 Multistainer-Coverslipper (Germany), and antigens were retrieved using citrate buffer (10 mM citric acid (#251275, Sigma)) with 0.05% Tween 20 (#P1379, Sigma), pH 6.0). The following-up procedures were performed using abcam mouse and rabbit specific HRP/DAB detection IHC kit (#ab64264, abcam) according to the manufacturer's instructions. Afterwards, the nuclei were stained using hematoxylin by Leica ST5020-CV5030 Multistainer-Coverslipper (Germany). Finally, the slides were scanned with Hamamatsu digital slide scanner NanoZoomer S60 (#C13210-01, Hamamatsu, Japan). IHC scores were determined based on percentage scores and intensity scores 25.

### Statistical analysis

All quantifications were obtained from at least 3 independent experiments or for animal experiments, at least 5 mice for each group. Data were presented as means ± SD. Statistical significance was assessed through one-way analysis of variance (ANOVA) with Tukey's test or Student's t-test in Python. Significance labels: *, *P* < 0.05; **, *P* < 0.01; ***, *P* < 0.001; ****, *P* < 0.0001; ns, not significant.

## Results

### SS treatment induced elevation of cellular ROS level, which enhanced malignant abilities of TNBC cells* in vitro* and *in vivo*

**Figure 1:**
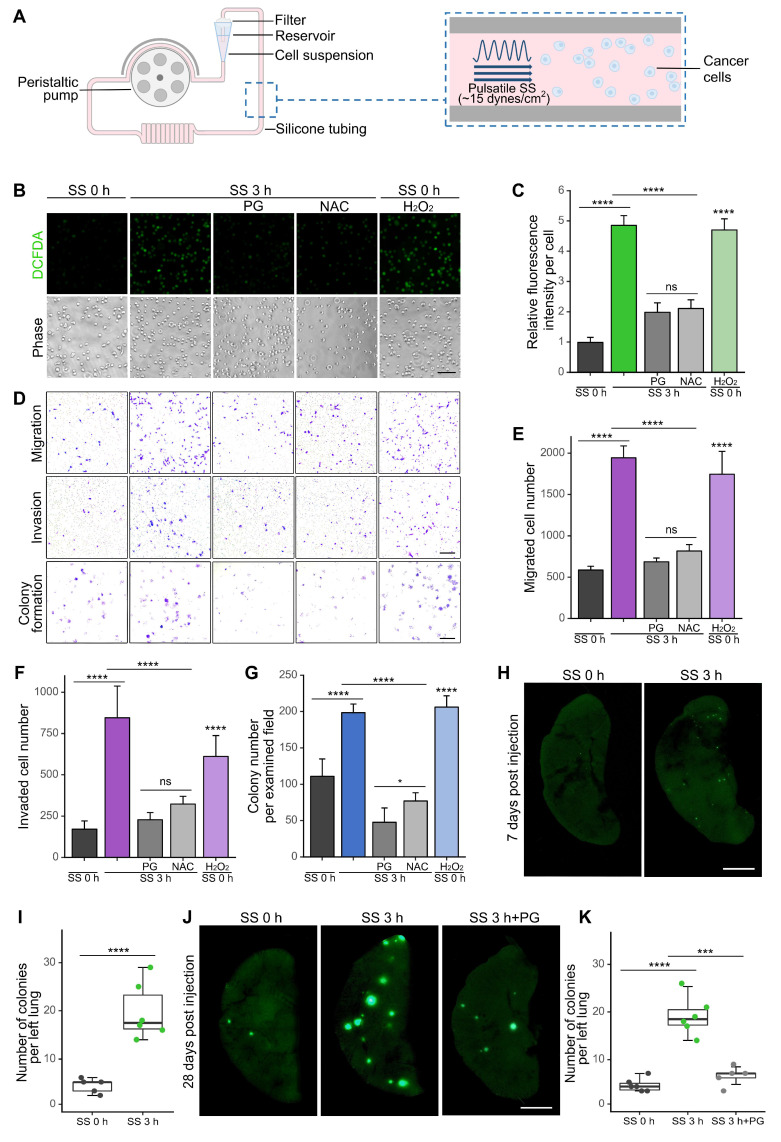
SS treatment elevated cellular ROS levels and enhanced invasiveness of TNBC cells *in vitro* and *in vivo*. (A) Schematic diagram of the engineered microfluidic system (adapted from previous work 15). The peristaltic pump generated pulsatile SS (approximately 15 dynes/cm^2^, representing the average arterial SS in human), together with the silicone tubing, mimicked the environment of cancer cells in circulation. Filter minimized air-borne contaminations and culture media evaporation. Cells were injected into the system through the reservoir at a density of 20 × 10^4^ cells/mL. (B-C) Representative images and quantifications of cellular ROS levels in MDA-MB-231 cells under indicated conditions: SS 0 h, no SS treatment; SS 3 h, SS 3 h+PG/NAC, SS for 3 h with/without 1-h pre-treatment and co-circulation with 20 μM PG or 5 mM NAC; SS 0 h+H_2_O_2_, pre-treatment with 100 μM H_2_O_2_ for 3 h, without SS. Cells were stained with 5 μM CM-H_2_-DCFDA for 15 min. Scale bar, 100 μm. (D-G) Representative images and quantified results of migration, invasion and colony formation assays of MDA-MB-231 cells under indicated conditions. For migration and invasion assays, 10^4^ cells were seeded and allowed to migrate or invade for 18 h. For colony formation assay, 1,000 cells were seeded and allowed to grow for 7 days. Scale bar, 200 μm for migration and invasion, 2 mm for colony formation. (H-I) Representative images and quantified results of left lung colonies of MDA-MB-231-GFP cells (with/without SS for 3 h) 7 days after tail vein injection. (J-K) Representative images and quantifications of left lung colonies of MDA-MB-231 cells (with/without SS for 3 h and pre-treatment and co-circulation with 20 μM PG) 28 days after tail vein injection. 30 × 10^4^ cells were injected into each NOD/SCID mouse (n ≥ 5 mice). Scale bar, 2 mm. The quantifications represent the means ± SD for three independent experiments. Significance was determined by t-test (I) or one-way ANOVA with Tukey's test (others). * *P* < 0.05, *** *P* < 0.001, **** *P* < 0.0001, ns, not significant.

To evaluate the impact of SS on cancer cells, we applied our microfluidic system which generated pulsatile SS and mimicked the arterial environment at an SS level of 15 dynes/cm^2^ (Figure 1A) 12,15. Consistent with our previous findings 15,16, SS treatment triggered a gradual increase of ROS levels that plateaued at 3 h in MDA-MB-231 cells Figure S1A-B) and similar trend was observed in the changes of migration ability (Figure S1A, C), indicating the quick response of cancer cells to SS stimulation. Treating cells with 3-h SS or 3-h SS plus 12-h recovery incubation (termed SS 3+12 h) did not affect cell viability (Figure S1D). SS treatment for 3 h was therefore selected as the proximal condition for later experiments. To explore whether ROS were indeed involved in SS-induced malignancy, antioxidants propyl gallate (PG) and N-acetyl-cysteine (NAC) were applied to our experimental conditions together with hydrogen peroxide (H_2_O_2_). As expected, PG and NAC demonstrated effectiveness in scavenging SS-induced ROS, from 4.9-fold down to 2.0-fold, while H_2_O_2_ caused an increase of ROS even without SS stimulation in MDA-MB-231 cells (Figure 1B-C). SS treatment also enhanced migration, invasion and colony formation to 3.3-fold, 4.9-fold and 1.8-fold, respectively, while scavenging ROS significantly suppressed these abilities without affecting cell viability (Figure 1D-G, Figure S4. Meanwhile, these abilities were enhanced by H_2_O_2_ in adherent cells (Figure 1D-G). Similar phenotypic observations were found in another TNBC cell line, BT549 Figure S2, suggesting they were not cell line-specific.

To further investigate whether SS could increase* in vivo* metastatic abilities, we injected SS-treated MDA-MB-231-GFP cells (MDA-MB-231 cells expressing green fluorescent protein) into non-obese diabetes/severe combined immunodeficiency (NOD/SCID) mice through tail vein. Notably, SS treatment resulted in a 4.7-fold increase in early lung micro-metastases 7 days post-injection (Figure 1H-I) and a 4.4-fold increase of later colonization 28 days post-injection, while the SS-enhanced colonization was significantly reduced when the elevation of ROS was inhibited by PG (Figure 1J-K). Together, these results indicated the contributions of SS-induced ROS to the enhancement of metastasis *in vitro* and* in vivo*.

### SS stimulation led to ROS-dependent expression of activator protein-1 (AP-1) family members FOSB, FOS, ATF3 and phosphorylation of cFOS, cJUN in TNBC cells

**Figure 2:**
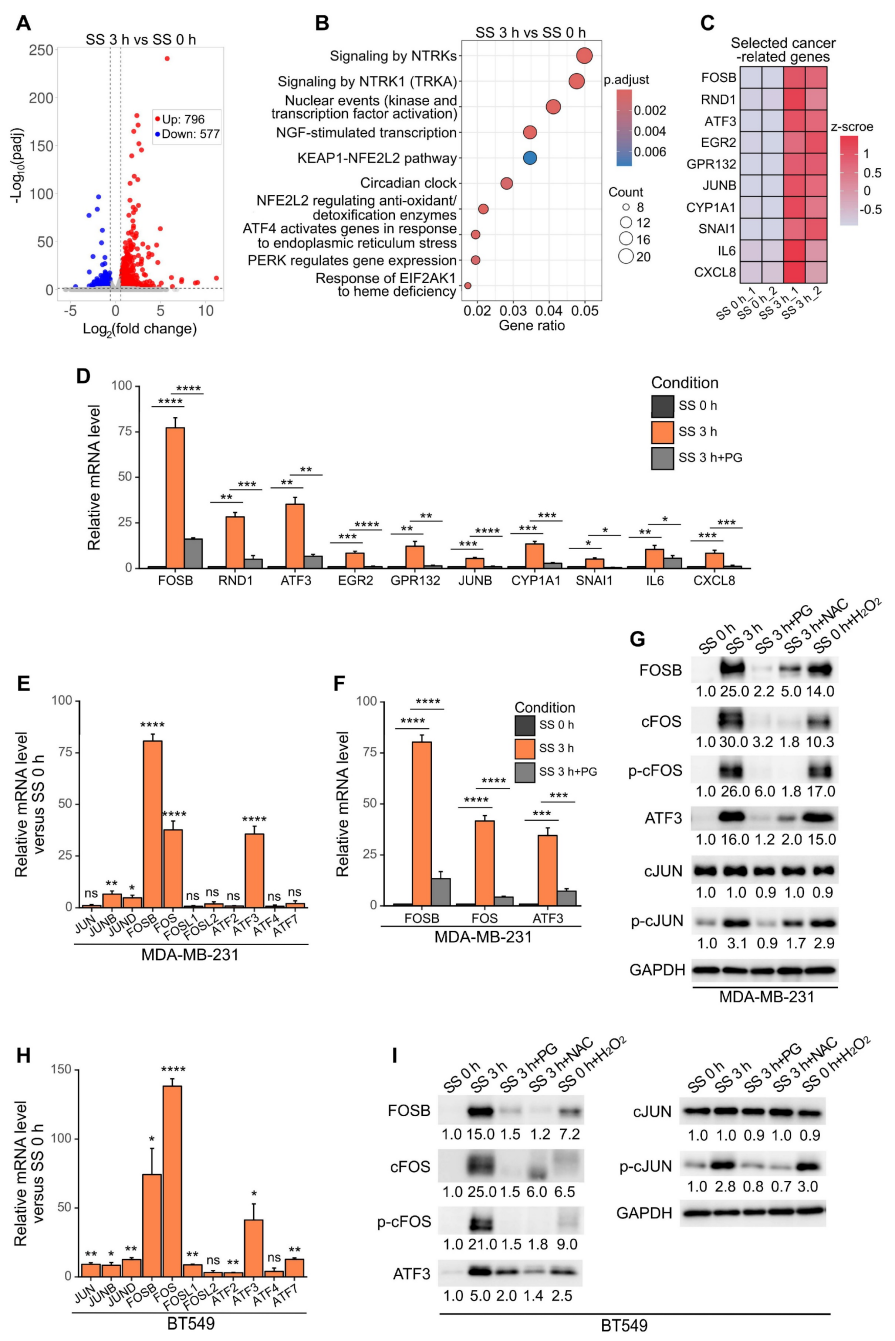
SS-induced ROS increased the expression of FOSB, cFOS, ATF3 and phosphorylation of cFOS and cJUN in multiple TNBC cell lines. (A) Volcano plot of differentially expressed genes (DEGs) after SS for 3 h compared to SS 0 h in MDA-MB-231 cells. Threshold was set as padj < 0.05, fold change > 1.5 for up-regulated genes and < 0.67 for down-regulated genes. (B) Reactome enrichment analysis of up-regulated DEGs after SS for 3 h compared to SS 0 h. (C) Heatmap of the selected top 10 DEGs with reported cancer-related function. (D) Validation of mRNA level increase and ROS-dependency of the selected genes from (C) using qPCR. (E) Relative mRNA levels of AP-1 family members (including JUN, FOS and ATF families) in MDA-MB-231 cells after SS for 3 h compared to SS 0 h determined by qPCR. (F) qPCR results showing the relative mRNA levels of FOSB, FOS and ATF3 in MDA-MB-231 cells under indicated conditions. (G) Western blotting of FOSB, cFOS, p-cFOS, ATF3, cJUN and p-cJUN in MDA-MB-231 cells under indicated conditions. (H) Relative mRNA levels of AP-1 members in BT549 cells after SS for 3 h compared to SS 0 h determined by qPCR. (I) Western blotting of selected proteins in BT549 cells under indicated conditions. The quantifications represent the means (± SD) for three independent experiments. Significance was determined by t-test (E, H) or one-way ANOVA with Tukey's test (D, F). * *P* < 0.05, ** *P* < 0.01, *** *P* < 0.001, **** *P* < 0.0001, ns, not significant.

To explore the effects of SS on the transcriptomics of cells, RNA-sequencing was performed to compare between the expression profile of MDA-MB-231 cells before and after SS treatment. Differential gene analysis identified 796 up-regulated genes and 577 down-regulated genes, within the range where adjusted *P* (padj) < 0.05, fold change > 1.5 or < 0.67 (Figure 2A). Reactome enrichment analysis 26 suggested that the up-regulated genes were enriched in transcription-related pathways (Figure 2B). Based on these, 10 top up-regulated genes which have demonstrated cancer-related function in published literature, namely FOSB, RND1, ATF3, EGR2, GPR132, JUNB, CYP1A1, SNAI1, IL6 and CXCL8 27-34, were selected, and their SS-ROS-dependent increase of mRNA level was validated using qPCR (Figure 2C-D). We realized that three out of the 10 selected genes, FOSB, ATF3 and JUNB, were from the activator protein-1 (AP-1) transcription factor family. Moreover, with the notion that the AP-1 family contains many members which function as dimers and their exact roles in cancer depend on different dimer composition 27, we further tested the mRNA levels of other AP-1 family members to obtain more information on their SS-induced signature. Consistent with our RNA-sequencing results, FOSB and ATF3 showed dramatic mRNA increase, to 80- and 34-fold, respectively (Figure 2E). Surprisingly, FOS demonstrated 42-fold increase transcriptionally after SS and this was counteracted by PG (Figure 2E-F), indicating it was also SS-ROS-inducible. At protein level, FOSB, cFOS (encoded by FOS), ATF3, and phosphorylated cFOS (p-cFOS) and cJUN (p-cJUN) were elevated to 25.0-, 30.0-, 16.0-, 26.0- and 3.1-fold, respectively, after SS treatment (Figure 2G). These increases were suppressed by PG and NAC, and could be induced by H_2_O_2_ (Figure 2G), suggesting the involvement of ROS. Given that the phosphorylation of cFOS and cJUN is required for their transcriptional activity 35, these findings suggested that SS and ROS promoted the activation of AP-1. Similar trends were observed in BT549 cells (Figure 2H-I), indicating the shared features of SS-ROS-induced gene signatures between two TNBC cell lines. Considering the dramatic mRNA and protein increase during SS, FOSB, FOS and ATF3 were therefore prioritized for further investigations.

### SS-ROS-induced AP-1 members were predominantly localized to the nucleus

**Figure 3:**
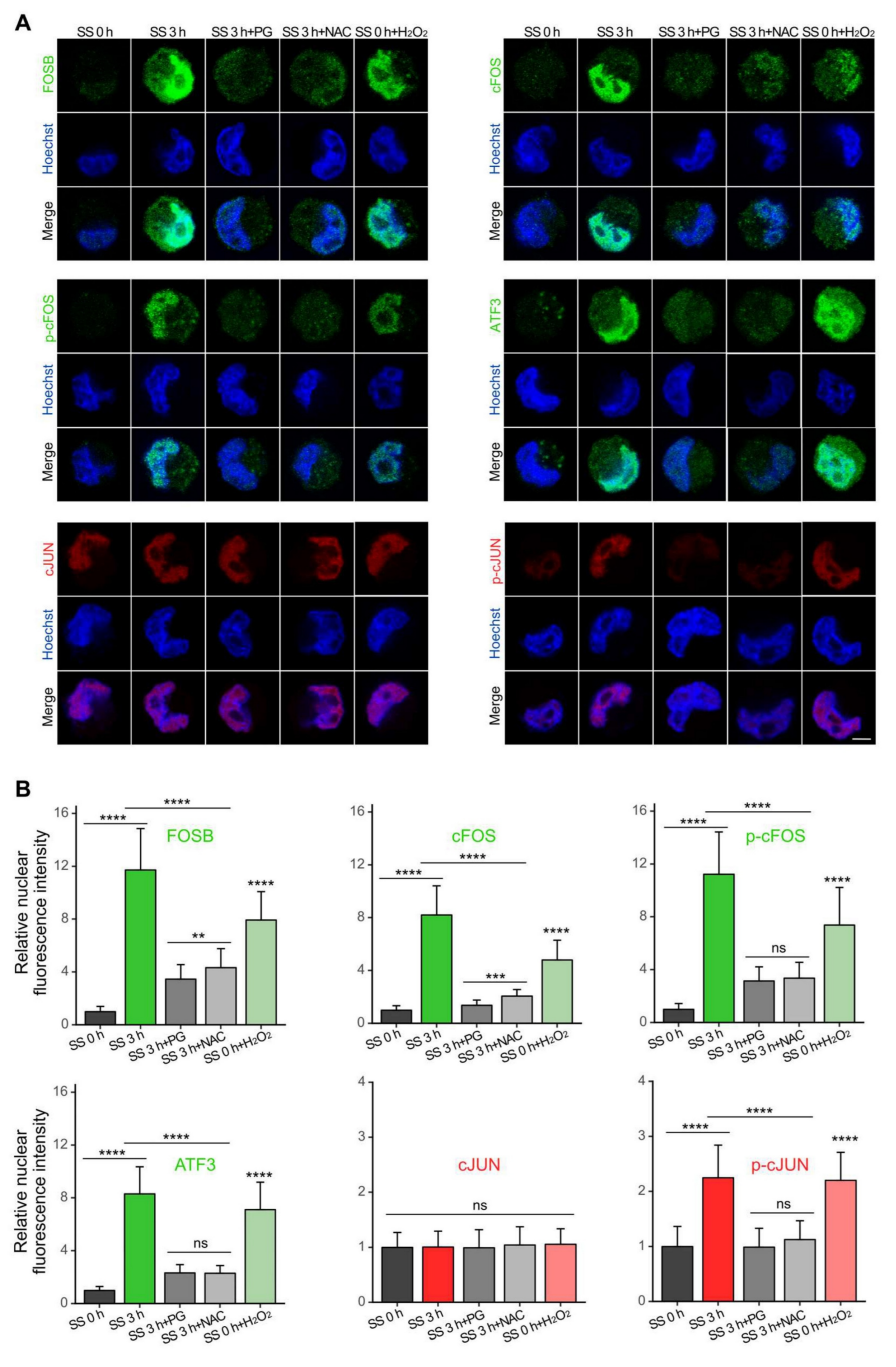
SS-ROS-induced FOSB, cFOS, p-cFOS, ATF3 and p-cJUN were predominantly localized to the nucleus. (A-B) Representative images and quantifications of immunofluorescence of FOSB, cFOS, p-cFOS, ATF3, cJUN and p-cJUN in MDA-MB-231 cells under indicated conditions. Scale bar, 5 μm. The quantifications represent the means ± SD for three independent experiments (n ≥ 100 cells). Significance was determined by one-way ANOVA with Tukey's test. ** P < 0.01, *** *P* < 0.001, **** *P* < 0.0001, ns, not significant.

As the functionality of transcription factors requires nuclear localization 36, we then examined the cellular distribution of the selected AP-1 proteins. Immunofluorescence results showed that, consistent with our earlier findings, SS treatment caused increase of protein levels of FOSB, cFOS, p-cFOS, ATF3 and p-cJUN, which were counteracted by PG and NAC (Figure 3). H_2_O_2_ treatment led to a p-cJUN increase comparable to that induced by SS, along with a moderate but significant elevation of FOSB, cFOS, p-cFOS, ATF3 (Figure 3), indicating that ROS production was necessary in this process. Moreover, these proteins were predominantly localized to the nucleus (Figure 3A), suggesting their potential transcriptional activity on downstream target genes.

### FOSB, FOS and ATF3 were responsible for SS-induced metastasis *in vitro* and *in vivo*

**Figure 4:**
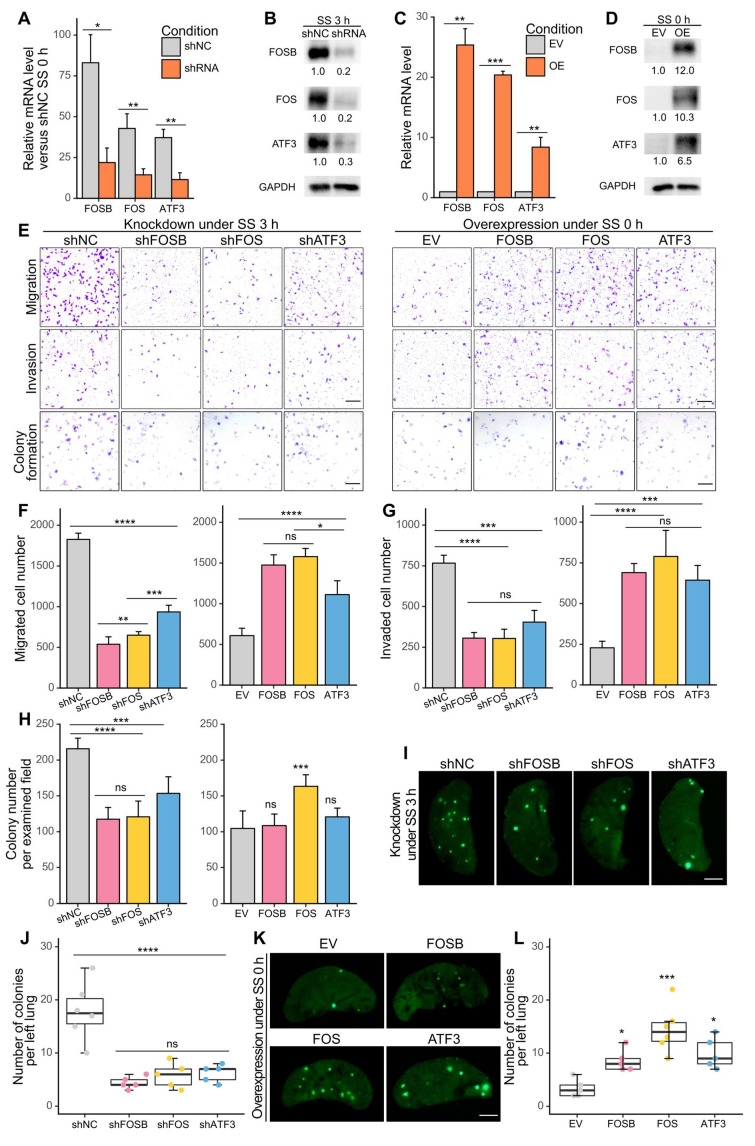
FOSB, FOS and ATF3 were associated with SS-induced malignant abilities *in vitro* and *in vivo*. (A-D) qPCR and Western blotting showing the knockdown (A-B) and overexpression (C-D) efficiency in MDA-MB-231-GFP cells after knocking down or overexpressing FOSB, FOS or ATF3. shNC, sh negative control; EV, empty vector. (E-H) Representative images and quantified results of migration, invasion and colony formation assays of the knockdown or overexpression cell lines. Scale bar, 200 μm for migration and invasion, 2 mm for colony formation. (I-L) Representative images and quantifications of left lung colonies of the knockdown (I-J) or overexpression (K-L) cell lines 28 days after tail vein injection (n ≥ 5 mice). For tail vein injection, 30 × 10^4^ cells were injected into each NOD/SCID mouse. Scale bar, 2 mm. The quantifications represent the means (± SD) for three independent experiments. Significance was determined by t-test (A, C) or one-way ANOVA with Tukey's test (F-H, J, L). * *P* < 0.05, ** *P* < 0.01, *** *P* < 0.001, **** *P* < 0.0001, ns, not significant.

To determine whether the selected AP-1 members contributed to SS-induced metastasis, we first performed targeted knockdown of FOSB, FOS and ATF3 in MDA-MB-231 cells using designed siRNAs Figure S3A-B) (23. Notably, knockdown of these genes caused dramatic reduction of migration, invasion and colony formation abilities under SS, by 50-80%, 70-80%, and 30-50%, respectively Figure S3C-F), preliminarily indicating their involvement during SS-induced malignancy. To achieve a long-term knockdown effect and to further test the function of the selected genes, we knocked down them using shRNAs and also overexpressed them in MDA-MB-231-GFP cells, and validated their efficiencies at both mRNA and protein levels (Figure 4A-D). Migration, invasion, colony formation assays were then performed to assess the metastatic features of the generated cell lines, where knockdown of FOSB, FOS and ATF3 significantly reduced their invasive abilities* in vitro* under SS (Figure 4E-H, left panels). Overexpressing any of these genes was strong enough to induce migration and invasion even without SS treatment, whereas only overexpression of FOS contributed to increased colony formation ability *in vitro* (Figure 4E-H, right panels), indicating their roles in SS-induced invasiveness. Next, to explore whether these genes were also involved in metastasis* in vivo*, we injected the SS-treated knockdown and untreated overexpression cell lines into NOD/SCID mice through tail vein. The results showed that knockdown of FOSB, FOS or ATF3 caused significant reduction of lung colonies formed by SS-treated cells 28 days post-injection (Figure 4I-J), while overexpression of them in non-treated cells increased lung colonization (Figure 4K-L). Interestingly, cells overexpressing FOS exhibited the strongest ability to colonize in the lung, compared to those overexpressing FOSB and ATF3 (Figure 4K-L). Taken together, these results demonstrated the involvement of AP-1 members FOSB, FOS, ATF3 in SS-induced metastasis, with FOS exhibiting the strongest metastasis-promoting potential among them.

### Overexpression of FOS, ATF3 and FOSB contributed to spontaneous metastasis

**Figure 5:**
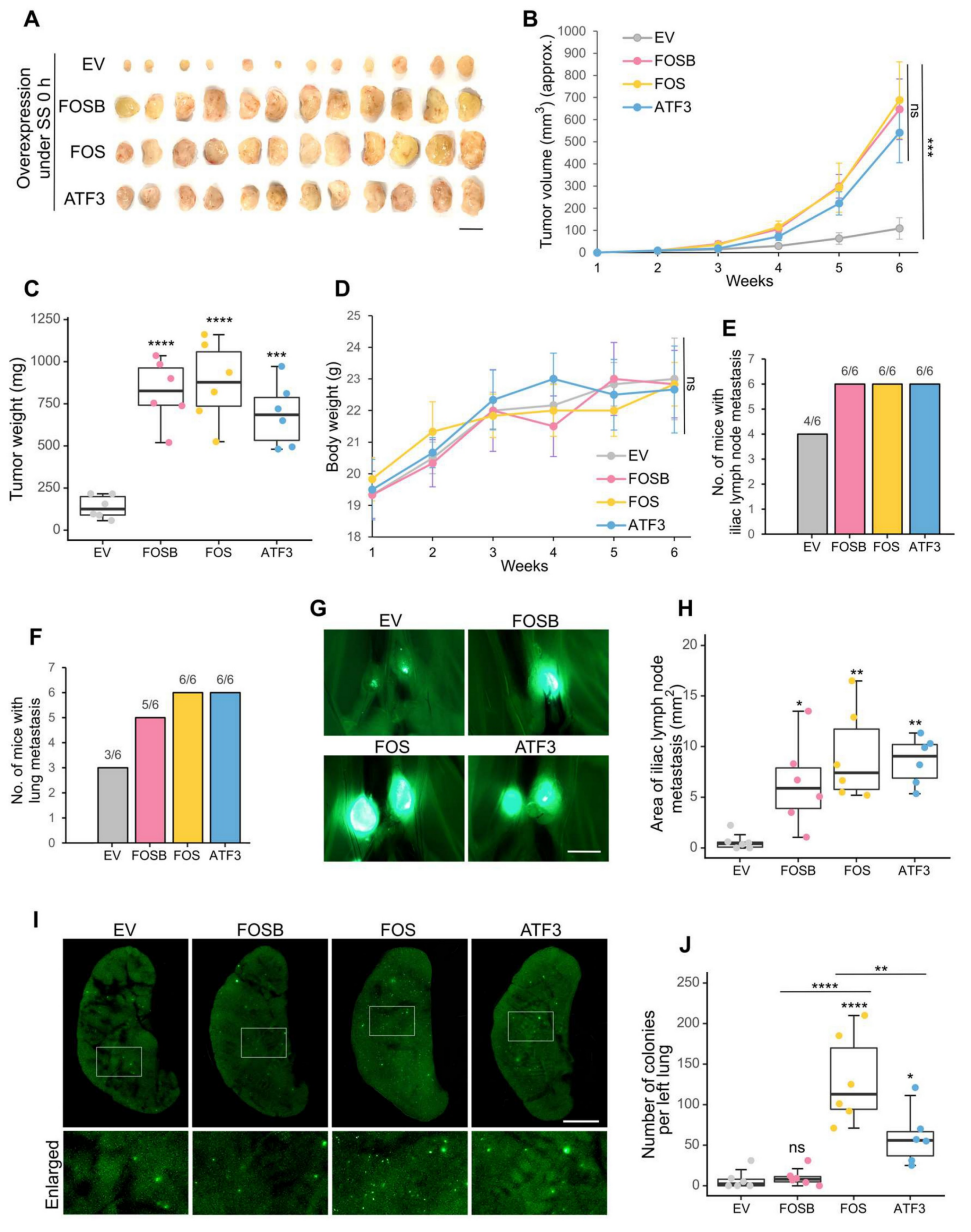
FOSB, FOS and ATF3 contributed to* in vivo* spontaneous metastasis. (A-C) Images and quantifications of primary tumors of MDA-MB-231-GFP cells overexpressing FOSB, FOS or ATF3 6 weeks after implantation into mammary fat pads of NOD/SCID mice (n = 6 mice). For orthotopic injection, 400 × 10^4^ cells were injected into each mouse. Scale bar, 1 cm. (D) Body weight of mice during the observed period. (E-F) The frequency of metastasis in the iliac lymph node and lung was quantified. (G-H) Representative images and quantified areas of iliac lymph node metastasis. Scale bar, 2 mm. (I-J) Representative images and quantifications of metastatic colonies in the lung. Scale bar, 2 mm. The quantifications represent the means ± SD from 6 mice. Significance was determined by one-way ANOVA with Tukey's test (B-D, H, J). * *P* < 0.05, ** *P* < 0.01, *** *P* < 0.001, **** *P* < 0.0001, ns, not significant.

After observing the metastasis-promoting effects of FOSB, FOS and ATF3 under SS, we then explored whether they were also associated with spontaneous metastasis, which is more complicated than the lung colonization model. To achieve this, the constructed overexpression cell lines were injected orthotopically into the mammary fat pads of NOD/SCID mice. Notably, they demonstrated significantly stronger tumor formation ability, displaying 8- to 10-fold increase of tumor weight 6 weeks post-injection (Figure 5A-C). Mice weight showed no significant difference among different groups (Figure 5D). In terms of metastasis, increased frequency of iliac lymph node and lung metastasis was observed (Figure 5E-F). The metastatic area in the iliac lymph nodes was also larger in the overexpression groups (Figure 5G-H). More importantly, overexpression of FOS resulted in the most dramatic increase of distant lung metastasis (on average 12-fold), which was followed by overexpression of ATF3 (on average 6-fold), whereas overexpression of FOSB showed much weaker effect (Figure 5I-J). These observations suggested the association between FOSB, FOS, ATF3 and spontaneous close-site metastasis and the particularly strong contributions of FOS to distant lung metastasis. Consequently, FOS was selected for further study as the most critical candidate.

### High expression of FOS was correlated with worse clinical outcomes

**Figure 6:**
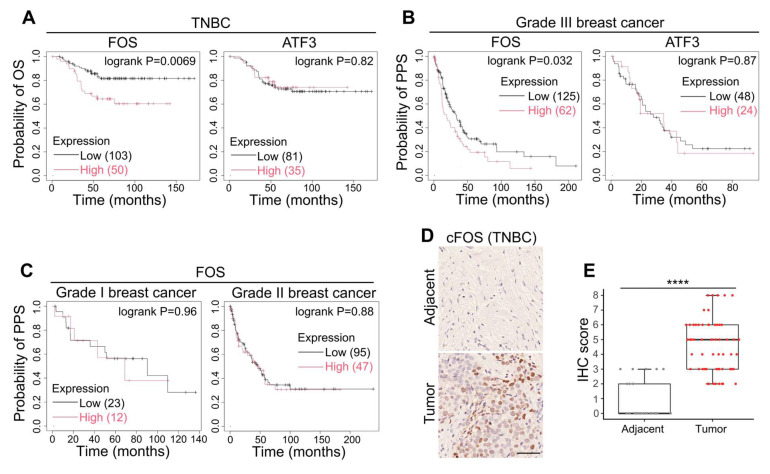
Clinical significance of FOS. (A) Kaplan-Meier plots of the OS of TNBC patients with references to the levels of FOS and ATF3. Parameters were set as: split patients by upper tertile; ER- (IHC/array), PR- (IHC), HER2- (array). (B-C) Kaplan-Meier plots of the PPS of Grade III breast cancer patients with references to the levels of FOS and ATF3 (B), or PPS of Grade I and II breast cancer patients with reference to FOS level (C). Parameters were set as: split patients by upper tertile; Grade I, II or III. (D-E) Representative images and quantifications of IHC on clinical TNBC samples (adjacent tissues, n = 29; tumor tissues, n = 60) against FOS. IHC scores were determined based on percentage scores and intensity scores 25. Scale bar, 50 μm. Significance was determined by t-test (E). **** *P* < 0.0001.

After observing the tumorigenic and metastasis-promoting roles of FOS and ATF3, we applied Kaplan-Meier analysis (24 to examine their correlation with clinical outcomes in patients. The results showed that high levels of FOS was correlated to shorter overall survival (OS) in TNBC patients and shorter post-progression survival (PPS) in Grade III breast cancer patients, whereas no significance was found between ATF3 level, OS and PPS (Figure 6A-B). Interestingly, FOS expression demonstrated minimal association with PPS of Grade I or II breast cancer (slower growth and less aggressive than Grade III) (Figure 6C), suggesting the critical role of FOS in advanced, highly metastatic breast cancer. Immunohistochemistry (IHC) of clinical TNBC samples also showed elevated level of cFOS in tumor tissues compared to adjacent non-tumor tissues (Figure 6D-E). Together, these observations suggested the clinical significance of FOS in tumorigenesis and cancer progression.

### Calcium channels acted as the frontline to initiate the SS-ROS-cFOS cascade

**Figure 7:**
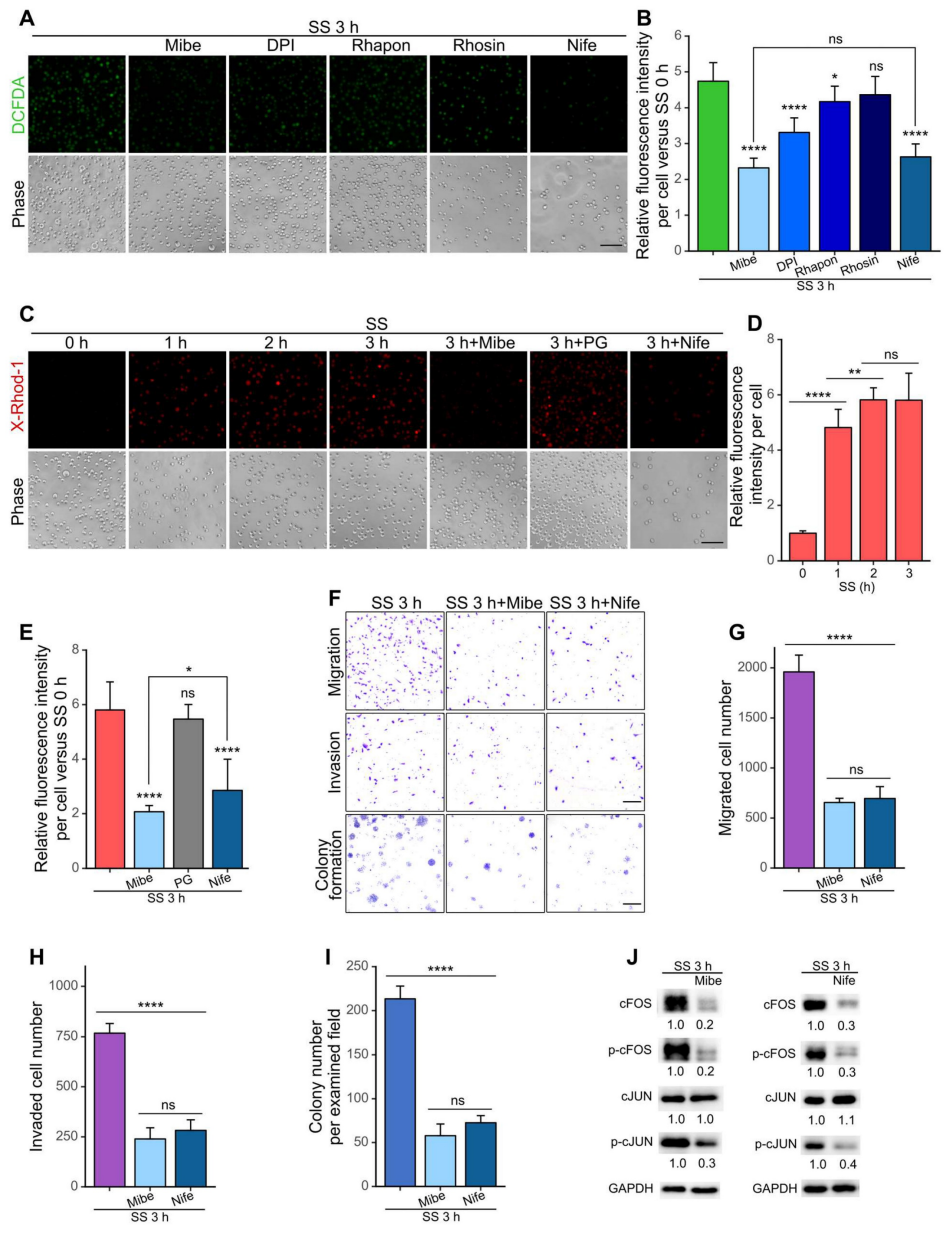
Calcium channel served as the mechano-transducer upon SS to initiate the ROS-cFOS/cJUN-metastasis axis. (A-B) Representative images and quantified results of cellular ROS levels. Cells were pre-treated for 1 h and co-circulated with or without 20 μM Mibe or 100 μM Nife (calcium channel blockers), 10 μM DPI (selective inhibitor of NADPH oxidase), 20 μM Rhapon (inhibitor of Cytochrome P450 family), or 30 μM Rhosin (inhibitor of Rho GTPases). Cells were stained with 5 μM CM-H_2_-DCFDA for 15 min. Scale bar, 100 μm. (C-E) Representative images and quantifications of cellular Ca^2+^ levels under indicated conditions. Cells were stained with 2 μM X-Rhod-1 for 15 min. Scale bar, 100 μm. (F-I) Representative images and quantified results of migration, invasion and colony formation assays under indicated conditions. Scale bar, 200 μm for migration and invasion, 2 mm for colony formation. (J) Western blotting showing the protein levels of cFOS, p-cFOS, cJUN and p-cJUN under indicated conditions. Relevant experiments were performed in MDA-MB-231 cells. The quantifications represent the means (± SD) for three independent experiments. Significance was determined by t-test (D) or one-way ANOVA with Tukey's test (B, E, G-I). * *P* < 0.05, ** *P* < 0.01, **** *P* < 0.0001, ns, not significant.

To further investigate how the SS-ROS-FOS-metastasis axis was initiated, a series of inhibitors targeting potential mechano-sensors and ROS producers were employed. Based on literature research, we selected calcium channels and RND1, a Rho GTPase with increased mRNA level after SS (Figure 2C-D) as the candidate targets, which have demonstrated mechano-sensing and cancer-related function 20,37-39. Moreover, we also chose CYP1A1 (cytochrome P450 family member 1A1) whose mRNA level was increased (Figure 2C-D), and NADPH oxidase (NOX), both of which represent important non-mitochondrial sources of ROS 18. For validation, we treated MDA-MB-231 cells with the calcium channel blocker Mibefradil (Mibe), Rho GTPase inhibitor Rhosin, CYP1A1 inhibitor Rhapontigenin (Rhapon) or NOX inhibitor DPI during circulation, and examined the cellular ROS levels. Interestingly, among all inhibitors selected, Mibe exhibited the strongest effect in reducing SS-triggered ROS (from 4.7- to 2.3-fold), followed by DPI (to 3.3-fold) and Rhapon (to 4.2-fold), whereas Rhosin showed no obvious effect (Figure 7A-B). These indicated the potential involvement of calcium channels, NOX and CYP450 enzymes in the upregulation of ROS by SS, and calcium channels were then prioritized for later investigations.

Time-course examination showed a rapid and dramatic increase of cellular Ca^2+^ levels after 1-h SS treatment and a further moderate increase at 2 h (Figure 7C-D). Such Ca^2+^ increase was quicker than ROS elevation which gradually increased and reached the plateau at SS 3 h Figure S1A-B). Moreover, as expected, the addition of Mibe but not PG significantly reduced cellular Ca^2+^ level, suggesting that the Ca^2+^ increase was due to calcium influx and that calcium channel activation was likely upstream of ROS elevation (Figure 7C, E). Importantly, in the presence of Mibe, the migration, invasion and colony formation abilities of MDA-MB-231 cells were dramatically reduced by 70% (Figure 7F-I), and the protein levels of cFOS, p-cFOS and p-cJUN decreased by nearly 80% (Figure 7J). We then selected another calcium channel blocker, Nifedipine (Nife), also a clinically applied drug for treating hypertension (40, and examined its effects on cancer cells under SS conditions. Interestingly, Nife treatment led to significant reduction of ROS, Ca^2+^ levels, migration, invasion, colony formation abilities and protein levels of cFOS, p-cFOS and p-cJUN in SS-stimulated MDA-MB-231 cells (Figure 7). Moreover, suppressing the function of FOS or calcium channels did not exert significant effect on cell viability upon SS treatment Figure S4, indicating that their major metastasis-promoting roles were enhancing invasiveness. To eliminate cell line-specific situations, we also tested the effects of Mibe and Nife on BT549 cells under SS, and observed similar trends Figure S5. Together, these findings suggested that calcium channels served as an essential frontline mechano-transducer to initiate the SS-induced malignancy cascade in TNBC cells through a SS-Ca^2+^-ROS-cFOS cascade.

### SS-induced ROS activated MAPK-FOS signaling to promote expression of invasion- and proliferation-related genes

**Figure 8:**
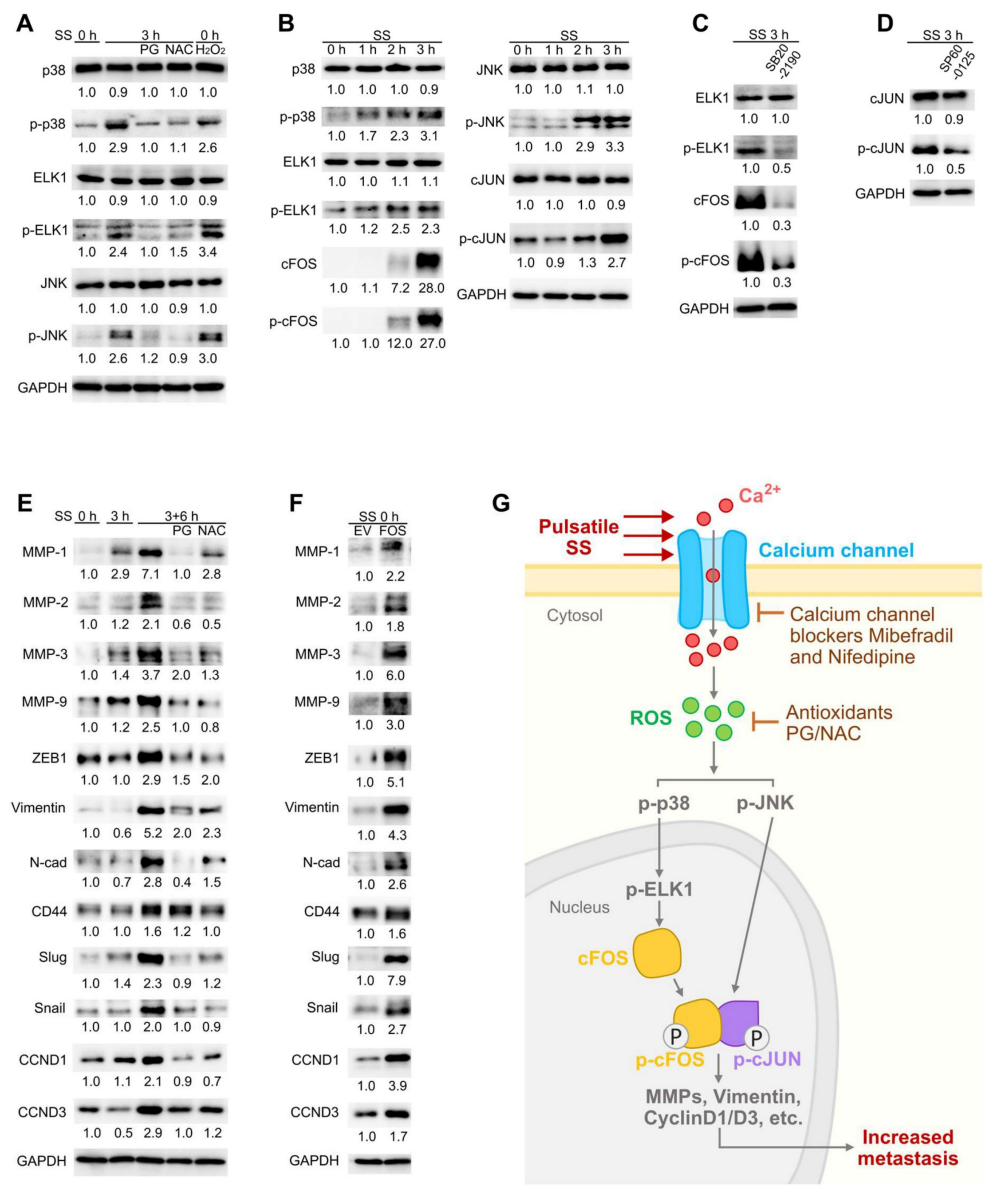
SS-induced ROS activated cFOS/cJUN through MAPKs and further promoted expression of downstream invasiveness and proliferation-related genes. (A-B) Western blotting of selected MAPKs, ELK1 and p-ELK1 (transcription factor of FOS) under indicated conditions. (C-D) Western blotting showing the levels of selected proteins under SS, with/without pre-treatment and co-circulation with 20 μM p38 inhibitor SB202190 (C) or 20 μM JNK inhibitor SP600125 (D). (E-F) Western blotting of metastasis-related proteins that were potentially downstream of cFOS/cJUN (selected from hTFtarget), under indicated conditions (E, SS 3+6 h represented SS 3 h followed by 6-h normal culture) or in FOS-overexpressing cells (F). (G) Graphical summary of the proposed signaling pathway. Relevant experiments were performed in MDA-MB-231 cells. The quantifications represent the means from three independent experiments.

Noticing the importance of cFOS and p-cFOS/p-cJUN heterodimer 27, we then explored the signaling cascades around them to further understand the molecular mechanisms. Mitogen-activated protein kinases (MAPKs) have been reported to regulate AP-1 and in particular, p38 could regulate cFOS expression while JUN N-terminal kinase (JNK) contributed to phosphorylation of cJUN 27. Moreover, ELK1 acted as the transcription factor of cFOS and was activated by p-38 through phosphorylation 41. Therefore, we tested the protein levels of p38, ELK1, JNK and their phosphorylated forms under SS in MDA-MB-231 cells. The results showed increase of p-p38, p-ELK1 and p-JNK but not their total proteins to more than 2-fold after SS, which were reduced by PG and NAC and elevated by solely H_2_O_2_ treatment (Figure 8A), suggesting SS-ROS-induced activation of MAPK pathway. Time-course explorations demonstrated sequential elevation of p-p38-p-ELK1-p-cFOS, and also p-JNK-p-cJUN (Figure 8B). Importantly, after 3-h SS stimulation, more than 80% of MDA-MB-231 cells demonstrated significant cFOS protein level increase compared to those without SS treatment Figure S6, indicating that cFOS elevation was indeed induced by SS conditions. Meanwhile, to confirm the involvement of p38 and JNK during this process, we applied p38 inhibitor SB202190 which effectively reduced levels of p-ELK1, cFOS, p-cFOS, while JNK inhibitor SP600125 reduced p-cJUN level under SS treatment in MDA-MB-231 cells (Figure 8C-D). These results suggested that SS regulated AP-1 activity via ROS-p-p38-p-ELK1-cFOS/p-cFOS and ROS-p-JNK-p-cJUN pathways.

Next, the downstream molecules of cFOS/cJUN that facilitated the metastasis of circulating TNBC cells were investigated. Metastasis involved a series of essential invasion and proliferation-related genes such as matrix metalloproteinases (MMPs) and cyclinDs (CCNDs) 42,43. With references to the human Transcription Factor target database (hTFtarget) 44 and other literatures, we selected MMPs-1, 2, 3, 9, ZEB1 (transcription factor of vimentin), vimentin, N-cadherin (N-cad), CD44, slug, snail, CCND1 and CCND3 as possible downstream targets, and examined their levels especially at SS 3+6 h (SS for 3 h followed by 6-h normal culture to allow downstream gene expression). Notably, SS caused strong increase of MMP-1, MMP-3 and vimentin to 7.1-, 3.7- and 5.2-fold, respectively, while the levels of MMP-2, MMP-9, ZEB1, N-cad, slug, snail, CCND1 and CCND3 were increased to more than 2-fold after 6 h post-SS (Figure 8E). As expected, they were reduced in the presence of PG and NAC (Figure 8E), suggesting the dependency on ROS elevation. To explore whether the expression of these genes could be induced by FOS, we examined these selected proteins in FOS-overexpressing cells, and observed increase of these molecules (Figure 8F). Taken together, these findings indicated that the SS-Ca^2+^-ROS-MAPKs-p-cFOS/p-cJUN axis drove the expression of malignancy-related genes which enhanced metastatic abilities of TNBC cells (Figure 8G).

## Discussion

Although SS could induce cell death in some circumstances, emerging evidence has been suggesting its metastasis-promoting potential which attracted our attention 7,8,16,17. Previous investigations by our colleagues found that SS at 15 dynes/cm^2^ for 6 h increased migration, adhesion, *in vivo* extravasation in zebrafish (*Danio rerio*) of MDA-MB-231 (and derived) cells in ROS-dependent manner and identified p-ERK and p-FAK as the key molecules involved 16,17. In this study, we provided more detailed information regarding how SS induced metastatic abilities of TNBC cells and found the involvement of calcium channel-ROS-MAPKs-FOS axis. To our knowledge, we applied SS-treated cells directly to mammalian model to assess their *in vivo* metastatic abilities for the first time, where we injected SS-stimulated cells into mice immediately after collecting them from our engineered circulatory system, and demonstrated their enhanced ability of forming micro-metastasis after 7 days and later macro-metastasis after 28 days *in vivo*. These findings provide solid evidence in support of the powerful malignancy-promoting potential of SS *in vivo*.

It is worth noticing that, similar to SS, ROS displayed complicated roles in cancer, either inducing cell death by accumulation of toxic ROS subtypes and consequently oxidative stress, or promoting metastasis as essential signaling molecules 45-47. However, some cancer cells demonstrated strong anti-oxidative ability or could convert toxic ROS to tumorigenic sub-types, thereby rendering them resistance to oxidative stress-inducing drugs 15,17,47. Interestingly and consistent with literature 16,17, our findings also revealed the strong dependency of SS-induced metastasis on cellular ROS elevation, which again highlighted the importance of ROS during cancer progression. More importantly, we also showed that the addition of antioxidants effectively scavenged cellular ROS under SS conditions and consequently, reduced formation of *in vivo* metastasis. These provide supportive evidence for targeting the redox status to treat against metastasis.

The roles of AP-1 transcription factors in cancer have been considered as a doble-edged sword due to their controversial performance 27. The AP-1 family consists of JUN, FOS, ATF and musculoaponeurotic fibrosarcoma (MAF) sub-families which function as dimers and exert both cancer-promoting and anti-cancer effects dependent on the dimer composition 27. While JUN family members can form homodimers, the other sub-families need to form heterodimers with JUN family proteins (e.g., the cFOS/cJUN dimer) 27,48. In our study, we observed that SS-induced ROS elevation led to huge increase in the expression of AP-1 members FOSB, FOS, ATF3, phosphorylation of cFOS and cJUN, and strong nuclear localization in TNBC cells. Subsequent explorations showed the most dramatic effects of FOS in promoting metastasis upon SS stimulation and also spontaneous metastasis. Interestingly, existing knowledge revealed that a combination between cJUN and cFOS significantly increased their affinity to the target sequences and enhanced their transcriptional activity together with phosphorylation 35,48, suggesting the active involvement of cFOS/cJUN heterodimer in transcriptional regulation. More importantly, literatures have been reporting the metastasis-promoting function of FOS in various types of cancers, including colon, liver, pancreatic and bone cancer 49-52. Nevertheless, FOS exhibited controversial roles in metastasis of breast cancer: it was found to be associated with metastatic phenotype of breast cancer cell lines MCF7 and MDA-MB-231 53-56, meanwhile one study observed reduced invasion upon overexpression of FOS in MCF7 cells 57; apart from these, elevation of FOS in immune cells such as macrophages and neutrophils increased bone and lung metastasis respectively 58-60, suggesting the complicated function of FOS depending on certain cell types and conditions of the tumor microenvironment.

Moreover, the SS-inducible features of FOS have been demonstrated in multiple endothelial cell lines and HeLa cells 61-63, while some studies showed ROS-dependent expression of FOS in endothelial cells under shear flow, or in chondrocytes and cardiomyocytes 64-66. We here applied a circulation-mimicking system and to our knowledge, observed for the first time the dramatic ROS-dependent elevation of FOS in TNBC cells under pulsatile SS and demonstrated its strong contributions to both SS-induced metastasis and spontaneous metastasis. Consistent with literature 27,67, we also observed MAPK-regulated features of cFOS/cJUN activity where p38 and JNK were involved, and subsequently the expression of invasiveness-related genes was promoted. These strongly suggest FOS to be a promising therapeutic target against TNBC metastasis.

Furthermore, mechano-sensors serve as the frontline for sensing mechanical force and play very important roles in mechano-transduction 19. They have been well investigated in endothelial cells especially vascular endothelial cells for they were always exposed to fluidic SS 20. The major cellular mechano-sensors include ion channels (e.g., sodium, potassium and calcium channels), cell surface receptors (e.g., GPCRs) and specialized membrane structures (e.g., caveolae, adhesion molecules) 20. However, the types and function of mechano-sensors in cancer progression, particularly CTCs, remained obscure. In our study, we demonstrated the great contributions of intracellular calcium elevation to the enhancement of metastatic abilities of cancer cells under circulatory conditions, highlighting the critical role of calcium channels as frontline mechano-sensor during metastatic dissemination. Interestingly, existing knowledge has suggested the involvement of calcium channels in cancer cell survival, proliferation, migration and invasion in multiple cancer types including breast, lung, prostate, liver, pancreatic, ovarian and colon cancer, for they allowed cells to obtain Ca^2+^ which acted as essential signaling molecules 38,68-74; meanwhile, Ca^2+^ also modulates the tumor microenvironment by regulating angiogenesis and the function of immune cells 73,75. More importantly, enhanced activity of calcium channels is associated with increased bone metastasis especially in breast cancer, where Ca^2+^ promotes not only the appearance of malignant phenotypes in tumor cells, but also osteoclast differentiation which in turn breaks the bone to facilitate metastasis, leading to poor clinical outcomes 74,76.

Notably, our observation that calcium channel blockers Mibe and Nife effectively reduced *in vitro* metastatic abilities of TNBC cells suggested the potential of calcium channel blockers as anti-metastatic strategy. Interestingly, Nife inhibited tumorogenesis and liver metastasis of colorectal cancer in mouse model 77. Anti-cancer effects of Mibe were also observed, where it suppressed proliferation of TNBC, glioblastoma and leukemia cells, and effectively treated one certain patient-derived pancreatic cancer xenograft 78-81. Moreover, Nife and Amlodipine (another clinically used calcium channel blocker) exhibited synergistic inhibition with cisplatin or gefitinib on the tumorigenesis and metastasis of head, neck, testis cancer or lung cancer, respectively 82,83, while application of Lercanidipine (a third-generation calcium channel blocker) significantly reduced viability of neuroblastoma, breast and prostate cancer cells, and by combining it with cisplatin, the cytotoxicity was further increased, suggesting synergistic effects of the two drugs to increase chemo-sensitivity 84. Taken together, these findings propose the critical function of calcium channel in metastasis, highlighting the potential of calcium channel blockers as anti-cancer and anti-metastasis strategies.

Interestingly, our study showed that the anti-metastatic impact of Mibe and Nife upon SS stimulation was likely an indirect effect of subsequent ROS level reduction, suggesting the interplay between Ca^2+^ influx and cellular ROS production. Our previous work reported that during SS treatment, the ROS were partially produced by the mitochondria 15, the major source of cellular ROS 85, while literature suggests that cellular Ca^2+^ increase could lead to mitochondrial Ca^2+^ uptake from the cytoplasm, further promoting mitochondrial ROS production under disease conditions 86. Moreover, elevation of cellular ROS and subsequent changes in the redox status could in turn regulate the activity of calcium channels 85,87. In this study, the calcium influx was not affected by scavenging ROS with PG, indicating the activation of calcium channels were more likely to be the upstream of ROS production. Consequently, targeting calcium channels will act as early-stage intervention and will potentially be a convincing therapeutic strategy.

Although our microfluidic system well mimicked the circulatory condition of CTCs, it still remained distinct from the actual physiological conditions where various other types of cells including blood cells and immune cells were involved. In our future investigations, we may consider these factors to better indicate the *in vivo* circulatory microenvironment. Notably, apart from the selected AP-1 members, our RNA-sequencing analysis also identified other interesting DEGs. For example, RND1, EGR2 and JUNB displayed dual roles in cancer progression, either promoting or suppressing metastasis 28,29,88-91, while others such as GPR132, CYP1A1 and SNAI1 have been reported to increase metastasis 30-32. Furthermore, our results suggested NOX and CYP450 enzymes as the potential ROS producers upon SS stimulation. Indeed, NOX, another major ROS producer, seats in the plasma membrane and has been reported responsive to SS 18,92. We also observed increase of mRNA level of CYP1A1, a member from the CYP450 family which mainly localized in the endoplasmic reticulum, indicating their SS-inducible activity in consistency with other studies using endothelial cells 93-95. Together, these suggest the complicated regulatory network within circulating cancer cells, consisting of interplay between SS, calcium channels, ROS producers, downstream signaling and the complex interactions among various cell types within the microenvironment, which deserves further investigation.

In summary, our results revealed that SS promoted metastasis of TNBC cells during circulation, dependent on calcium channel activity, ROS production and subsequent AP-1 activation. AP-1 transcription factor family members FOSB, FOS and ATF3 were involved in SS-induced invasiveness while FOS demonstrated the strongest impact in enhancing spontaneous metastasis. Upon SS, calcium channels in the plasma membrane acted as the frontline mechano-sensor and their activation caused Ca^2+^ influx, which then promoted the production of cellular ROS. Elevation of ROS further triggered expression of FOS and subsequent phosphorylation of cFOS/cJUN heterodimer through p38-ELK1/JNK cascade. Following up AP-1 activation, a series of downstream malignancy-associated genes such as MMPs, vimentin and CCNDs were upregulated, leading to the metastatic phenotypes. Our findings suggest a metastasis-promoting axis initiated by SS, and sequentially composed of calcium channels, ROS and cFOS, providing potential therapeutic targets for preventing and treating metastasis.

## Supplementary Material

Supplementary figures and tables.

## Data Availability

The data that support the findings of this study are available from the corresponding author upon reasonable request.

## Abbreviations

CTC: circulating tumor cells
SS: shear stress
ROS: reactive oxygen species
TNBC: triple-negative breast cancer
AP-1: activator protein-1
GPCR: G-protein coupled receptor
PG: propyl gallate
NAC: N-acetyl-L-cysteine
Mibe: Mibefradil dihydrochloride
DPI: diphenylene iodonium chloride
Rhapon: Rhapontigenin
Nife: Nifedipine
OS: overall survival
PPS: post-progression survival
IHC: Immunohistochemistry
MAPK: mitogen-activated protein kinase
JNK: JUN N-terminal kinase
MMP: matrix metalloproteinase
CCND: cyclinD
