# Supplementary material for: Shear Stress Promotes Metastasis of Triple-negative Breast Cancer Cells Through Calcium Channel-ROS-FOS Axis
Source: Int J Biol Sci. 2026 Apr 8;22(8):4315–33. doi: 10.7150/ijbs.127645 (PMC13137952; doi:10.7150/ijbs.127645)
Supplement: Supplementary file 1 — Supplementary figures and tables. [file ijbsv22p4315s1.pdf]

# Supporting Information

**Shear Stress Promotes Metastasis of Triple-negative Breast Cancer Cells Through Calcium Channel–ROS–FOS Axis**

Huaxing Xiong, Muya Zhou, Kathy Qian Luo*

Department of Biomedical Sciences, Faculty of Health Sciences

University of Macau

Taipa, Macao SAR 999078, China

* Kathy Qian Luo (corresponding author)

Ministry of Education Frontiers Science Center for Precision Oncology

University of Macau

Taipa, Macao SAR 999078, China

E-mail: kluo@um.edu.mo

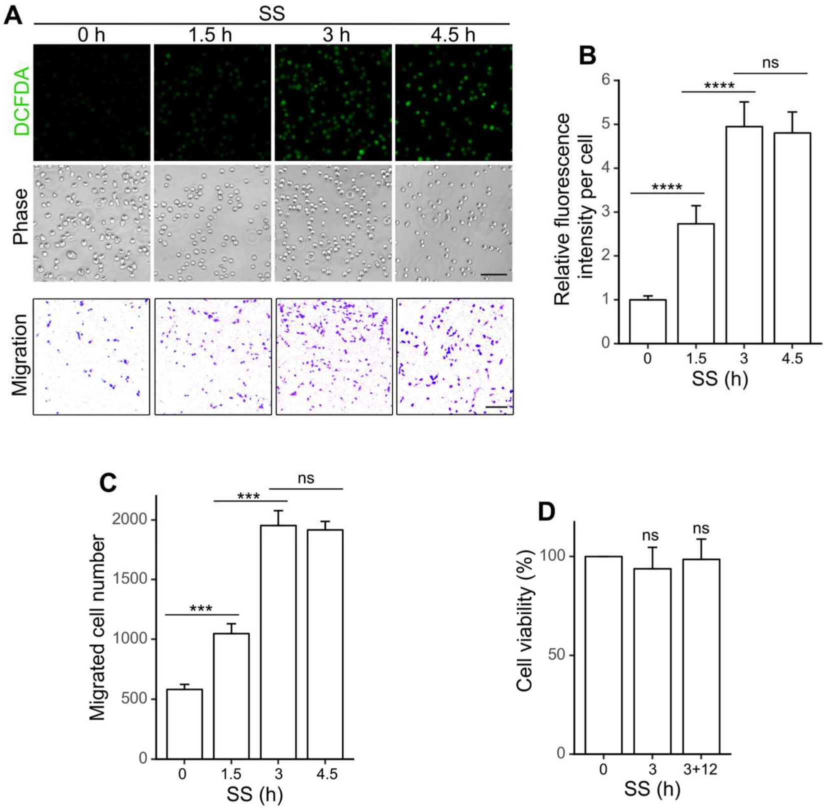


**Figure S1**. SS treatment induced ROS elevation and cell migration which reached a plateau at 3 h without affecting viability. (A-C) Representative images and quantifications of cellular ROS levels and migration assay in MDA-MB-231 cells under indicated conditions. For ROS detection, cells were stained with 5 μM CM-$H_2$-DCFDA for 15 min. For migration assay, $10^4$ cells were seeded. Scale bar, 100 μm for ROS, 200 μm for migration. (D) Quantified results of cell viability before and after 3-h SS treatment, or after 12-h recovery post-SS treatment (SS 3+12 h, for observing delayed cell death) by MTT assay. The quantifications represent the means ± SD for three independent experiments. Significance was determined by t-test. *** $P < 0.001$, **** $P < 0.0001$, ns, not significant.

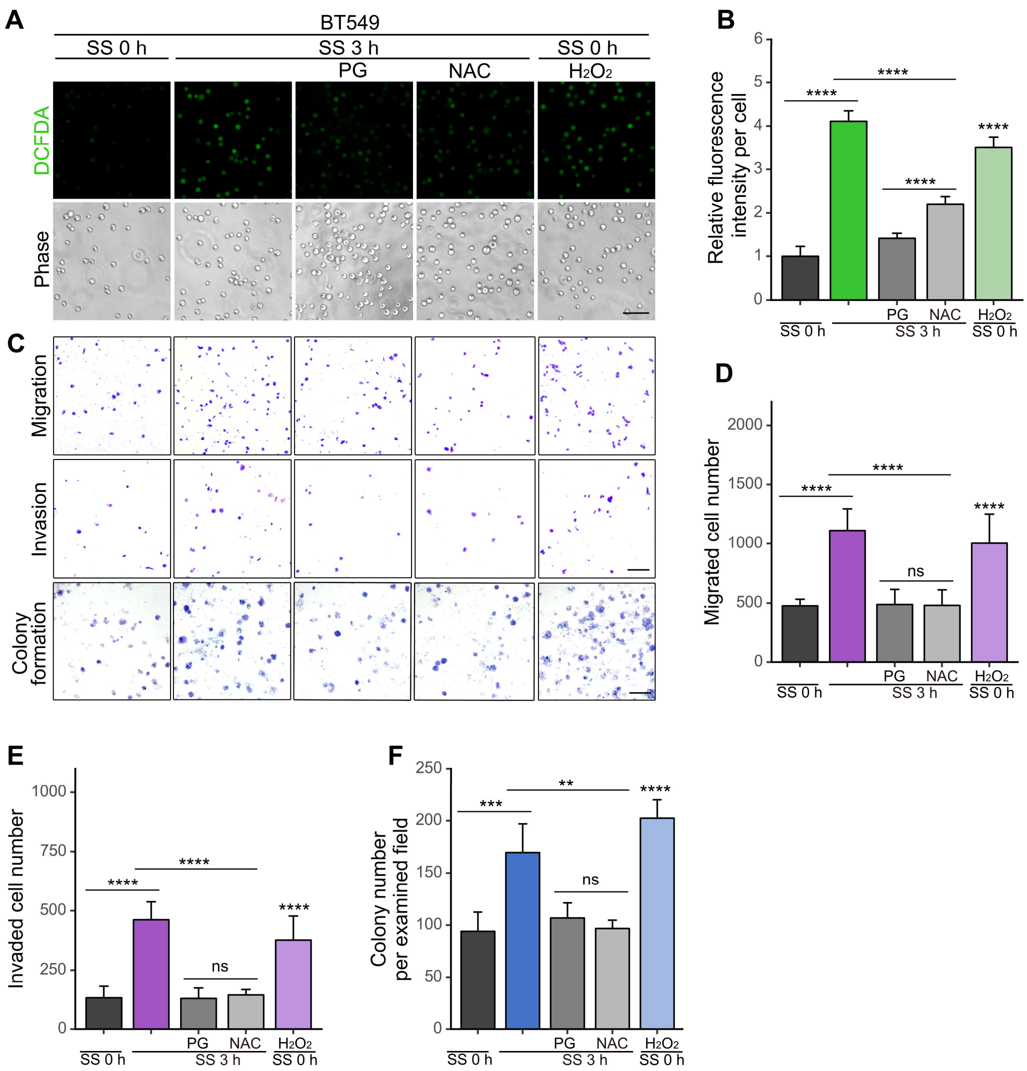


**Figure S2.** SS-induced ROS, migration, invasion and colony formation were also observed in BT549 cells. (A-B) Representative images and quantified results of cellular ROS levels in BT549 cells under indicated conditions: pre-treatment with 50 μM $H_2O_2$ for 3 h, other conditions were as described earlier. Scale bar, 100 μm. (C-F) Representative images and quantifications of migration, invasion and colony formation assays of BT549 cells under indicated conditions. For migration and invasion assays, 5,000 cells were seeded and allowed to migrate or invade for 18 h. For colony formation assay, 1,000 cells were seeded and allowed to grow for 10 days. Scale bar, 200 μm for migration and invasion, 2 mm for colony formation. The quantifications represent the means ± SD for three independent experiments. Significance was determined by one-way ANOVA with Tukey's test. ** $P < 0.01$, *** $P < 0.001$, **** $P < 0.0001$, ns, not significant.

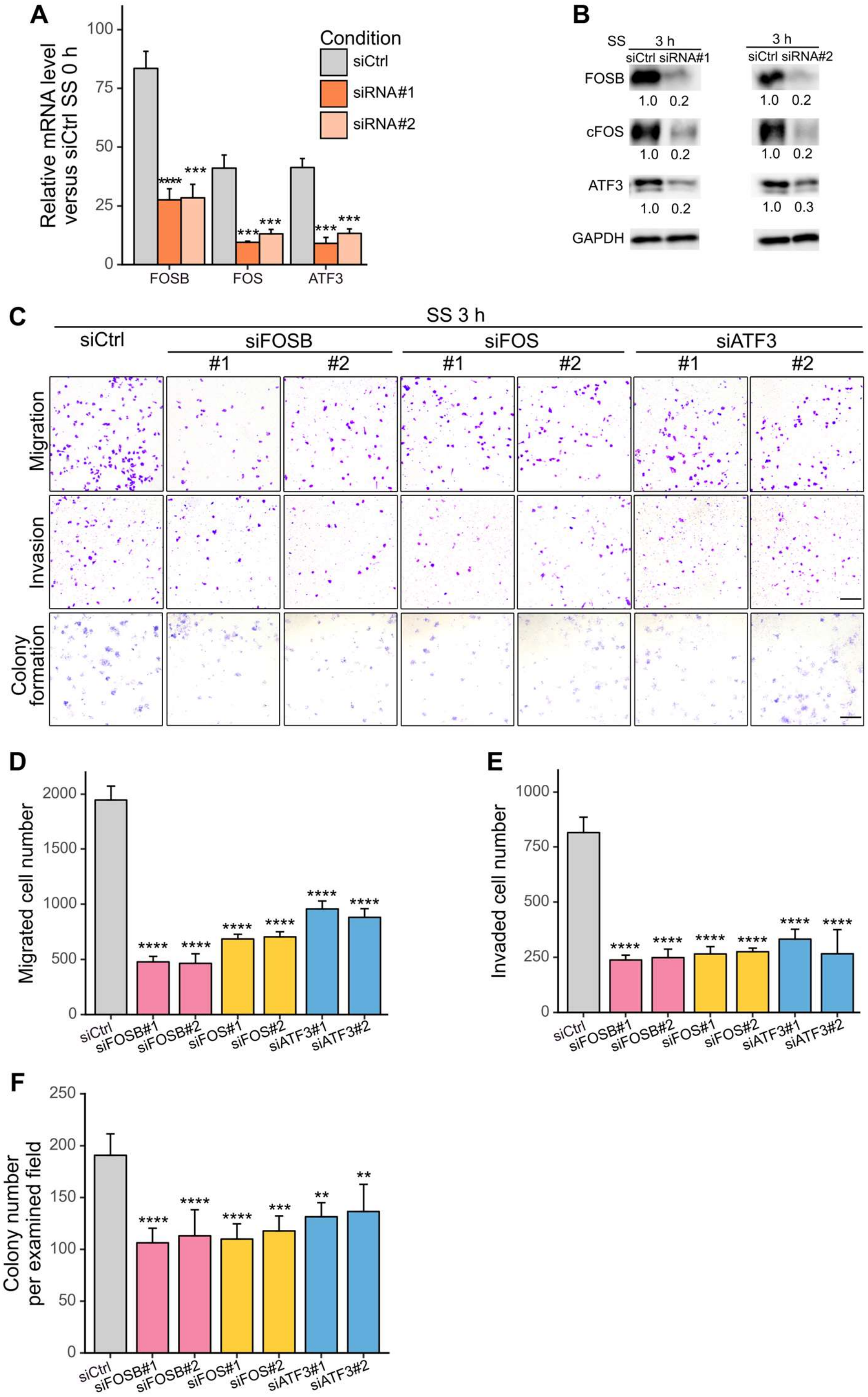


**Figure S3.** FOSB, FOS and ATF3 contributed to SS-induced metastatic abilities *in vitro*. (A-B) qPCR and Western blotting of knockdown efficiency in SS-treated MDA-MB-231 cells after knocking down FOSB, FOS or ATF3 using siRNAs. (C-F) Representative images and quantifications of migration, invasion and colony formation assays of siRNA-knocked-down cells. Scale bar, 200 μm for migration and invasion, 2 mm for colony formation. The quantifications represent the means (± SD) for three independent experiments. Significance was determined by one-way ANOVA with Tukey's test. ** $P < 0.01$, *** $P < 0.001$, **** $P < 0.0001$.

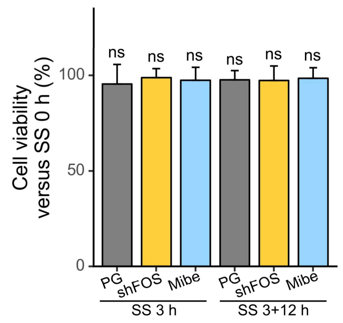


**Figure S4.** ROS, FOS and calcium channel activity were not responsible for cell survival under SS conditions. Quantified results of cell viability under SS treatment compared to no treatment by MTT assay, after scavenging ROS with PG, FOS knockdown or inhibiting calcium channels with Mibe. SS 3+12 h represented SS treatment for 3 h followed by 12-h culture for capturing delayed cell death events. The quantifications represent the means ± SD from three independent experiments. Significance was determined by t-test. ns, not significant.

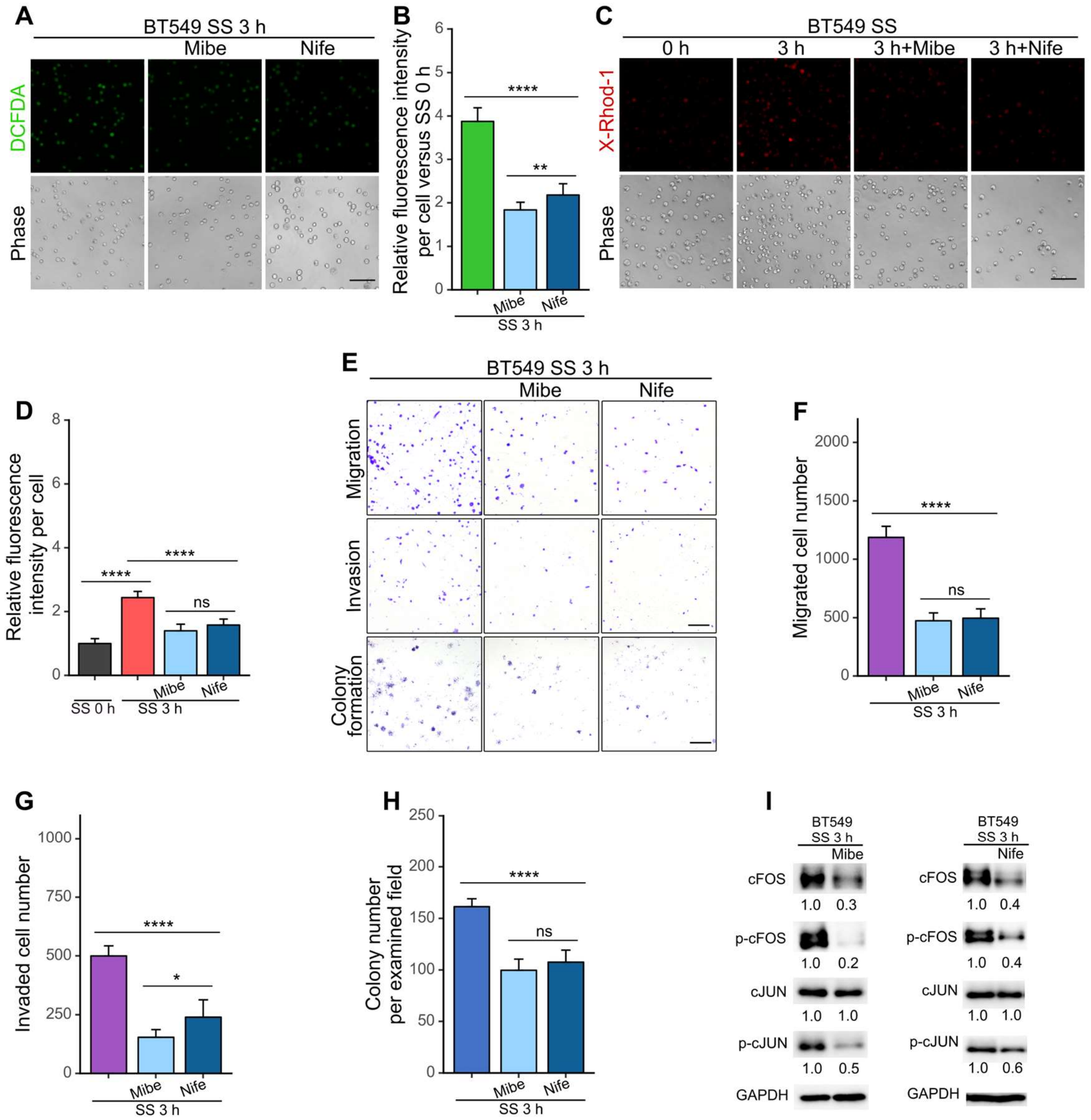


**Figure S5.** SS–calcium–ROS–metastasis axis was also functional in BT549 cells. (A-D) Representative images and quantified results of cellular ROS and $Ca^{2+}$ levels under SS, with

or without pre-treatment and co-circulation with 20 µM Mibe or 100 µM Nife. Cells were stained with 5 µM CM-$H_2$-DCFDA (for ROS) or 2 µM X-Rhod-1 (for $Ca^{2+}$) for 15 min. Scale bar, 100 µm. (E-H) Representative images and quantifications of migration, invasion and colony formation assays under indicated conditions. Scale bar, 200 µm for migration and invasion, 2 mm for colony formation. (I) Western blotting showing the protein levels of cFOS, p-cFOS, cJUN and p-cJUN under indicated conditions. Relevant experiments were performed in BT549 cells. The quantifications represent the means (± SD) for three independent experiments. Significance was determined by one-way ANOVA with Tukey's test (B, D, F-H). * $P < 0.05$, ** $P < 0.01$, **** $P < 0.0001$, ns, not significant.

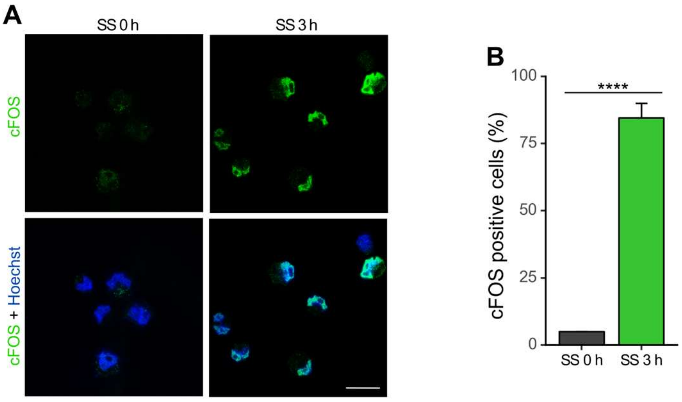


**Figure S6.** cFOS elevation was induced by SS treatment in a large proportion of cells. (A-B) Representative images and quantifications of immunofluorescence of cFOS in MDA-MB-231 cells before and after 3-h SS treatment. Scale bar, 20 µm. The 95$^{th}$ percentile of fluorescence intensity in untreated cells was set as the threshold for distinguishing cFOS positive and negative cells. The quantifications represent the means ± SD from three independent experiments (n ≥ 100 cells). Significance was determined by t-test. **** $P < 0.0001$.

## Supplementary tables

**Table S1.** List of primers for qPCR

| Gene name | Forward (5'-3') | Reverse (5'-3') |
|---|---|---|
| FOSB | GCTGCAAGATCCCCTACGAAG | ACGAAGAAGTGTACGAAGGGTT |
| RND1 | TTAGCGAAGGATTGCTATCCAGA | GTATCCCAGAGACTAAGCTCCA |
| ATF3 | CCTCTGCGCTGGAATCAGTC | TTCTTTCTCGTCGCCTCTTTTT |
| EGR2 | TCAACATTGACATGACTGGAGAG | AGTGAAGGTCTGGTTTCTAGGT |
| GPR132 | AAGGTGACCGCCTACATCTTC | GTCTTCCGTCTGGAACACCG |
| JUNB | ACGACTCATACACAGCTACGG | GCTCGGTTTCAGGAGTTTGTAGT |
| CYP1A1 | TCGGCCACGGAGTTTCTTC | GGTCAGCATGTGCCCAATCA |
| SNAI1 | TCGGAAGCCTAACTACAGCGA | AGATGAGCATTGGCAGCGAG |
| IL6 | ACTCACCTCTTCAGAACGAATTG | CCATCTTTGGAAGGTTCAGGTTG |

| | | |
|---|---|---|
| CXCL8 | TTTTGCCAAGGAGTGCTAAAGA | TTTTGCCAAGGAGTGCTAAAGA |
| JUN | TCCAAGTGCCGAAAAAGGAAG | CGAGTTCTGAGCTTTCAAGGT |
| JUND | TCATCATCCAGTCCAACGGG | TTCTGCTTGTGTAAATCCTCCAG |
| FOS | GGGGCAAGGTGGAACAGTTAT | CCGCTTGGAGTGTATCAGTCA |
| FOSL1 | CAGGCGGAGACTGACAAACTG | TCCTTCCGGGATTTTGCAGAT |
| FOSL2 | CAGAAATTCCGGGTAGATATGCC | GGTATGGGTTGGACATGGAGG |
| ATF2 | AATTGAGGAGCCTTCTGTTGTAG | CATCACTGGTAGTAGACTCTGGG |
| ATF4 | ATGACCGAAATGAGCTTCCTG | GCTGGAGAACCCATGAGGT |
| ATF7 | GAGACGACAGACCGTTTGTGT | AGGCGTTTGATCTGCAATGAT |
| GAPDH | CTGGGCTACACTGAGCACC | AAGTGGTCGTTGAGGGCAATG |

**Table S2.** List of Antibodies

| **Antibody name** | **Company** | **Catalog #** | **Application** |
|---|---|---|---|
| ATF3 | CST* | 18665 | WB (1:1000), IF (1:100) |
| CCND1 | CST | 55506 | WB (1:1000) |
| CCND3 | CST | 2936 | WB (1:1000) |
| CD44 | CST | 3570 | WB (1:1000) |
| ELK1 | CST | 9182 | WB (1:1000) |
| p-ELK1 (Ser383) | CST | 9181 | WB (1:1000) |
| cFOS | CST | 31254 | WB (1:1000), IF (1:100), IHC (1:20) |
| p-cFOS (Ser32) | CST | 5348 | WB (1:1000), IF (1:100) |
| FOSB | CST | 2251 | WB (1:1000), IF (1:100) |
| GAPDH | CST | 2118 | WB (1:1000) |
| JNK | CST | 9252 | WB (1:1000) |
| p-JNK (Thr183/Tyr185) | CST | 9251 | WB (1:1000) |
| cJUN | CST | 9165 | WB (1:1000), IF (1:100) |
| p-cJUN (Ser73) | CST | 3270 | WB (1:1000), IF (1:100) |
| MMP-1 | CST | 54376 | WB (1:1000) |
| MMP-2 | CST | 87809 | WB (1:1000) |
| MMP-3 | Abcam | Ab52915 | WB (1:1000) |
| MMP-9 | CST | 13667 | WB (1:1000) |
| N-cadherin | CST | 13116 | WB (1:1000) |
| p38 | CST | 8690 | WB (1:1000) |
| p-p38 | CST | 4511 | WB (1:1000) |
| Slug | CST | 9585 | WB (1:1000) |
| Snail | CST | 3879 | WB (1:1000) |
| Vimentin | CST | 5741 | WB (1:1000) |
| ZEB1 | Sigma | 90510 | WB (1:1000) |

| | | | |
|---|---|---|---|
| Goat anti-Rabbit IgG (H+L) Secondary Antibody, Alexa Fluor Plus 488 | Invitrogen | A11034 | IF (1:100) |
| Goat anti-Rabbit IgG (H+L) Secondary Antibody, Alexa Fluor Plus 594 | Invitrogen | A11037 | IF (1:100) |
| Goat anti-Rabbit IgG (H+L)-HRP Secondary Antibody | Bio-Rad | 1706515 | WB (1:5000) |
| Goat anti-Mouse IgG (H+L)-HRP Secondary Antibody | Bio-Rad | 1706516 | WB (1:5000) |

*CST: Cell Signaling Technology

**Table S3.** List of siRNAs

| siRNA name | Target sequence (5'-3') |
|---|---|
| siFOSB#1 | UGUUAACCCUUCGUACACUUCTT |
| siFOSB#2 | UGCAAGAUCCCCUACGAAGAGTT |
| siFOS#1 | UCCAGAAGAAGAAGAGAAAAGTT |
| siFOS#2 | UUCAUUAUUGGAAUUAACCUGTT |
| siATF3#1 | AUAUUACUUAUUUAUCCUAGUTT |
| si ATF3#2 | CUGUCAGAAUAAUAAAUAUUGTT |
| shCtrl | UUCUCCGAACGUGUCACGUTT |

**Table S4.** List of shRNAs

| shRNA name | TRC Number | Target sequence (5'-3') |
|---|---|---|
| shFOSB | | UGUUAACCCUUCGUACACUUCTT |
| shFOS | | UCCAGAAGAAGAAGAGAAAAGTT |
| shATF3 | | AUAUUACUUAUUUAUCCUAGUTT |
| shNC | SHC002 | CAACAAGATGAAGAGCACCAA |

**Table S5.** List of overexpression vectors

| Vector name | Transcript ID | Sequence length |
|---|---|---|
| FOSB | NM_006732.3 | 1017 bp |
| FOS | NM_005252.4 | 1188 bp |
| ATF3 | NM_001674.4 | 1917 bp |
